# Effect of welding fume on heart rate variability among workers with respirators in a shipyard

**DOI:** 10.1038/srep34158

**Published:** 2016-09-28

**Authors:** Bor-Cheng Han, I-Jung Liu, Hsiao-Chi Chuang, Chih-Hong Pan, Kai-Jen Chuang

**Affiliations:** 1 School of Public Health, College of Public Health and Nutrition, Taipei Medical University, Taipei, Taiwan; 2 Department of Nursing, Cardinal Tien College of Healthcare & Management, New Taipei City, Taiwan; 3 School of Respiratory Therapy, College of Medicine, Taipei Medical University, Taipei, Taiwan; 4 Division of Pulmonary Medicine, Department of Internal Medicine, Shuang Ho Hospital, Taipei Medical University, Taipei, Taiwan; 5 Institute of Labor, Occupational Safety and Health, Ministry of Labor, Taipei, Taiwan; 6 School of Public Health, National Defense Medical Center, Taipei, Taiwan; 7 Department of Public Health, School of Medicine, College of Medicine, Taipei Medical University, Taipei, Taiwan

## Abstract

Welding fume exposure is associated with heart rate variability (HRV) reduction. It is still unknown whether respirator can reduce effect of welding fume on HRV among welding workers in a shipyard. We recruited 68 welding workers with respirator and 52 welding workers without respirator to measure HRV indices, including standard deviation of normal-to-normal intervals (SDNN) and square root of the mean squared differences of successive intervals (r-MSSD) by ambulatory electrocardiographic (ECG). Personal exposure to particulate matter less than or equal to 2.5 μm in diameter (PM_2.5_) was measured by a dust monitor. The association between 5-minute mean PM_2.5_ and log_10_-transformed HRV indices was analyzed by mixed-effects models. We found 5-minute mean PM_2.5_ was associated with 8.9% and 10.3% decreases in SDNN and r-MSSD. Effect of PM_2.5_ on HRV indices was greatest among workers without respirator {SDNN: 12.4% (95% confidence interval = −18.8–−6.9); r-MSSD: 14.7% (95% confidence interval = −20.8–−8.6)}. Workers with respirator showed slight decreases in HRV indices {SDNN: 2.2% (95% confidence interval = −6.3–−1.9); r-MSSD: 4.0% (95% confidence interval = −6.4–−1.6)}. We conclude that respirator use reduces the effect of PM_2.5_ exposure on HRV among workers performing welding in a shipyard.

Welding is a fabrication process to join materials, such as metal, by intense heat. Electric welding by electrode is frequently used to improve the assembly of the larger metal pieces in shipyard. Welding fume, a complex mixture of metals, gases and particles, arises from the base metal, especially from electrodes while welding^1^. Previous studies have reported that welding workers are exposed to welding fumes generated during the welding process^2^. The majority of welding fume particles is found to be in coarse and fine size ranges by mass concentration^3^. Compared with coarse particles, fine particles {particulate matter (PM) less than or equal to 2.5 μm in diameter (PM_2.5_)} is often measured due to its ability to penetrate deep into the alveolar regions of the lung and induce adverse cardiovascular effects (4. Evidence from epidemiological studies suggest that heart rate variability (HRV) can be used as an early disease marker of PM-induced adverse cardiovascular effects^5^,^6^,^7^. Decrease in HRV has been reported to alter the heart’s ability to respond to external signals, leading to myocardial infarction and sudden cardiac death^8^.

Recent studies have shown that workers exposed to metal fume are associated with HRV changes. Magari *et al*.^9^ reported that exposure to high levels of PM_2.5_ was associated increased standard deviation of normal-to-normal intervals (SDNN) among a cohort of 39 boilermakers^9^. Cavallari *et al*.^10^ found that high concentrations of PM_2.5_ were associated with decreased night square root of the mean squared differences of successive intervals (r-MSSD) for 26 male boilermaker construction workers 10. Fan *et al*.^11^ observed that acute decline of HRV was associated with exposure to welding PM_2.5_ among 66 welders^11^. On the other hand, Scharrer *et al*.^12^ found no effect of clinical significance of a short-term high-level exposure to welding fumes on time- and frequency-domain parameters of HRV among 20 healthy individuals^12^. According to these studies, the findings on the association between welding particles and HRV changes are still inconsistent. Moreover, it is still unknown whether respirator can reduce adverse effect of welding fume on HRV.

In order to answer whether occupational exposure to welding fume is associated with impaired cardiac autonomic control and the effectiveness of respirator on protecting workers from welding fume, we recruited a panel of welding workers in Taiwan to clarify these scientific questions.

## Results

Summarized statistics for 120 participants’ characteristics, heart rate variability monitoring, PM_2.5_ exposure and climate conditions are presented in Table 1. The study participants were adult workers with a mean age of 51.1 years {standard deviation (SD) = 10.3 years}, 37.5% were current smokers, and 56.7% used respirator while working. There were no female workers among our population. No significant different was observed in age, BMI and seniority between workers with and without respirators. For PM_2.5_, temperature and humidity monitoring, no significant difference was observed in PM_2.5_ exposure and climate conditions between workers with and without respirators during the study period. For HRV monitoring, the workers with respirators had significantly higher values of HRV indices (Log_10_ SDNN and Log_10_ r-MSSD) than did the workers without respirators.

The results of associations between concurrent PM_2.5_ and HRV indices estimated by the mixed-effects models are shown in Tables 2. With age, BMI, smoking, temperature and relative humidity being adjusted in all mixed-effects models, we found that PM_2.5_ exposure was associated with HRV changes for welding workers in general. It is noteworthy that PM_2.5_ exposure significantly decreased HRV indices for workers without respirators. Log_10_ SDNN decreased by 12.4% per IQR of PM_2.5_ at 5-minute mean among workers without respirators. No significant association between PM2.5 and Log_10_ SDNN was observed among workers with respirators. Likewise, r-MSSD decreased by 14.7% per IQR of PM_2.5_ at 5-minute mean among workers without respirators but only decreased by 4.0% among workers with respirators.

Table 3 shows the results of associations of concurrent PM_2.5_ with HRV indices estimated by the mixed-effects models stratified by smoking. With age, BMI, temperature and relative humidity being adjusted in all mixed-effects models, we found that PM_2.5_ exposure was associated with HRV reduction for both smoking and non-smoking workers without respirators. No significant association of PM_2.5_ with HRV reduction was observed among workers with respirators except Log10 r-MSSD in smoking workers.

We further assessed effect modification in mixed-effects models and found an effect modification of PM_2.5_ by respirator usage (Table 4). Workers without respirators showed a decrease of 7.4% in Log_10_ SDNN, which was associated with an increased 5-minute mean PM_2.5_. In contrast, workers with respirators showed no significant change in Log_10_ SDNN. Likewise, workers without respirators showed a decrease of 10.2% in Log_10_ r-MSSD by PM_2.5_ exposure, but workers with respirators only showed a mild decrease of 3.5% in Log_10_ r-MSSD.

## Discussion

Welding fume has been commonly measured as total dust or inhalable particles in past studies. However, fine particles, which can reach the alveoli, are more specific with regard to cardiopulmonary diseases and systemic particles exposure from welding fume^14^. Previous studies demonstrate the association of environmental exposure to PM_2.5_ with decreased HRV in human subjects^5^,^6^,^7^. Recent studies further report the association between occupational exposure to PM_2.5_, mainly from welding, and HRV changes in workers^9^,^10^,^11^,^12^. The present study confirms that occupational exposure to PM_2.5_ has comparable magnitudes of effects on decreasing HRV indices in workers. Magari *et al*.^9^ showed that SDNN decreased by 1.4% per 1 mg/m^3^ of concurrent PM_2.5_ (−0.77 msec) among 39 boilermakers^9^. Cavallari *et al*.^10^ reported that total PM_2.5_ exposure was associated with decline in night r-MSSD by 18.8% (−0.006 msec/μg/m^3^) among 26 boilermakers^10^.

In addition to the PM_2.5_ effects on HRV indices, the present study shows respirator could modify the association between PM_2.5_ and HRV. As the results of mixed-effects model with effect modifier showed (Table 3), workers without respirators had higher effects of PM_2.5_ on HRV reduction compared to those in workers with respirators. This finding is the first one to demonstrate the protective effect of respirator on reducing PM_2.5_ exposure and cardiovascular adverse effects. Our finding also provides further evidence to support that respirator can be the last resort in controlling welding fume exposure to worker in the aspect of occupational safety and health management system^15^,^16^.

The possible pathophysiological mechanism between PM_2.5_ and autonomic alteration has been well discussed in previous studies. In brief, inhaled PM_2.5_ may exacerbate the autonomic function of the heart via induced inflammation in lung and proinflammatory cytokine expression in cardiac macrophages^17^. The time course of PM_2.5_ on HRV in workers at 5-minute mean is in agreement with the findings of previous studies among workers^18^, young adults^19^ and general population^20^. It has been reported that fine particles can affect both sympathetic and parasympathetic nervous systems immediately. The biological mechanism is PM-induced activation of pulmonary neural reflexes secondary to autonomic alteration after PM inhalation^21^,^22^,^23^.

Several limitations should be addressed in the present study. First, the measurement of PM_2.5_ might not be representative of welding fume exposure in this study because it was monitored by only personal dust monitor. There might be unmeasured gaseous pollutants that could contribute to the observed HRV reduction. However, if the present findings were driven by gaseous pollutants, there would be no effect modification by respirator status. Therefore, the observed HRV reduction was less likely due to gaseous pollutants as gases travel freely through respirators. Second, the unmeasured meteorological variables, such as wind speed and wind direction, were likely to influence the level of personal PM_2.5_ exposure^24^. Such limitation might lead to exposure misclassification and bias the pollution effects on HRV reduction toward null^25^. Third, we could not exclude the respiration’s confounding effects on the association of personal PM_2.5_ exposure with HRV reduction because our participants’ physical activities and breathing patterns were not measured in the present study^26^. Lastly, short-term reduction of HRV has not been associated with higher risk of cardiovascular disease clinically. Cohort study is needed to elucidate the effect of respirator on the relationship between welding fume and cardiovascular health.

## Conclusion

The present study found that personal exposure to welding fume was significantly associated with decreased HRV. Such adverse cardiovascular effect was lower among welders with respirators than among welders without respirators. The use of a respirator is therefore beneficial for occupational health management and welder health improvement. Therefore, we encourage employers to provide suitable respirators and adequate training for employees.

## Methods

### Ethics

The Ethics Committee of the Taipei Medical University-Joint Institutional Review Board approved the study protocol. The methods were carried out in accordance with the approved guidelines. All subjects received written and oral information prior to inclusion and provided informed consent.

### Study participants

We recruited 120 healthy male welding workers from a shipyard located in Taiwan as our study participants for this panel study in July and August in 2013 and 2014. The main duty of these welding workers was to weld all types of steel plates of varying sizes and shapes with electric welder. The company was obligated to provide respirators and training courses for all welding workers before they performed arc welding. The type of respirator was disposable activated-carbon facemask. We assigned a well-trained assistant to conduct eight-hour continuous personal PM_2.5_ exposure monitoring and HRV monitoring for each participant while working (from 09:00 am to 17:00 pm). Each participant’s age, body mass index (BMI), smoking, work history, current health status, respirator usage and work-activity pattern, including sitting, walking, talking, welding, dining, etc. were obtained from a questionnaire by a professionally trained nurse. None of participants had hyperthyroidism, hypoxemia, acute cardiopulmonary failure and paced cardiac rhythm. They were informed of the objective of this study, the benefits and possible danger to health. The ethic committee of the Taipei Medical University Joint Institutional Review Board (Taipei, Taiwan) approved this study (TMU-JIRB No.: 201306041). An informed consent was obtained from each participant before the study embarked and the individuals were allowed to give up at any moment.

### HRV monitoring

We performed continuous ambulatory electrocardiographic (ECG) monitoring for each participant while working by using a PacerCorder 3-channel device (model 461A; Del Mar Medical Systems LLC, Irvine, CA, USA) with a sampling rate of 250 Hz (4msec). All ECG tapes were analyzed by using a Delmar Avionics model Strata Scan 563 (Irvine, CA, USA). Each 5-minute segment of normal-to-normal (NN) intervals was taken for HRV analysis. The time domain measurements of HRV were SDNN and r-MSSD. Each participant obtained approximately 12 successful segments of 5-minute HRV measurements per hour for data analysis.

### Personal PM_2.5_ exposure monitoring

We conducted continuous monitoring of PM_2.5_ and meteorological conditions for each participant while working. PM_2.5_, temperature and relative humidity were measured continuously by a personal dust monitor (DUSTcheck Portable Dust Monitor, model 1.108; temperature and humidity sensor, model 1.153FH; Grimm Labortechnik Ltd., Ainring, Germany), which measured and recorded 1-min mass concentrations of PM_2.5_ as well as temperature and relative humidity in the breathing zone. After monitoring, the raw data for 1-min PM_2.5_, temperature and relative humidity were matched with the monitoring time of HRV for data analysis.

### Statistical analysis

We applied mixed-effects models to examine the association of PM_2.5_ with SDNN and r-MSSD for our study participants by running R statistical software version 2.15.1. The SDNN and r-MSSD were log_10_-transformed to improve normality and stabilize the variance. We treated each participant’s age, BMI, smoking (Yes vs. No), respirator usage as time-invariant variables, while 5-minute mean PM_2.5_, 5-minute mean temperature, 5-minute mean relative humidity, log_10_ SDNN and log_10_ r-MSSD as time-varying variables in our data analysis. The outcome variables were log_10_ SDNN and log_10_ r-MSSD, and the exposure variables were 5-minute mean PM_2.5_. We treated each participant as a random effect in our mixed-effects models. In further analyses, we investigated if PM_2.5_ effects differed among smokers and non-smokers using mixed-effects models stratified by smoking.

Effect modification by respirators usage (Yes vs. No) was assessed in a separate mixed model by including interaction terms between PM_2.5_ exposure and respirators usage among all workers. Minimizing Akaike’s Information Criterion (AIC)^13^ was used as the criteria of model selection. The pollution effects are expressed as percent changes in HRV by an interquartile range (IQR) change in pollution levels for PM_2.5_.

## Additional Information

**How to cite this article**: Han, B.-C. *et al*. Effect of welding fume on heart rate variability among workers with respirators in a shipyard. *Sci. Rep.* **6**, 34158; doi: 10.1038/srep34158 (2016).

## Figures and Tables

**Table 1:** Summary statistics for participants’ characteristics, and PM_2.5_ exposure, heart rate variability and climate conditions at 5-minute mean.

	All workers	With respirator	Without respirator	ANOVA	
				p-value	Scheffe’s test
Respirator usage, no					
Yes	68	68	0	—	—
No	52	0	52	—	—
Smoking, no					
Yes	45	25	20	—	—
No	75	43	32	—	—
Age, year					
Mean ± SD	51.1 ± 10.3	50.1 ± 9.7	52.4 ± 8.9	0.65	—
Range	26–61	26–59	30–61		
No	120	68	52		
BMI, kg/m^2^					
Mean ± SD	24.8 ± 2.4	23.8 ± 1.9	25.2 ± 2.6	0.72	—
Range	19.2–29.1	19.2–28.4	21.3–29.1		
No	120	68	52		
Seniority, year					
Mean ± SD	27.6 ± 12.5	27.2 ± 11.9	27.8 ± 12.1	0.82	—
Range	1–45	1–45	1–45		
No	120	68	52		
PM_2.5_, mg/m^3^					
Mean ± SD	1.3 ± 0.8	1.8 ± 1.1	1.1 ± 0.7	0.84	—
IQR	0.7	0.8	0.6		
Range	0.1–5.4	0.1–5.4	0.1–4.9		
No	11520	6528	4992		
Temperature, °C					
Mean ± SD	28.2 ± 3.4	27.7 ± 2.9	28.4 ± 3.1	0.72	—
Range	24.7–33.1	24.7–32.6	25.5–33.1		
No	11520	6528	4992		
Relative humidity, %					
Mean ± SD	74.2 ± 6.7	76.6 ± 5.9	68.2 ± 6.9	0.67	—
Range	52.1–78.4	52.1–78.4	52.7–75.3		
No	11520	6528	4992		
Log_10_ SDNN, msec					
Mean ± SD	1.64 ± 0.32	1.79 ± 0.17	1.51 ± 0.13	<0.05	With/Without
Range	0.89–2.11	0.99–2.56	0.89–2.11		
No	11520	6528	4992		
Log_10_ r-MSSD, msec					
Mean ± SD	1.51 ± 0.21	1.63 ± 0.15	1.39 ± 0.26	<0.05	With/Without
Range	0.49–2.07	0.52–1.91	0.49–2.07		
No	11520	6528	4992		

BMI, body mass index; PM_2.5_, particulate matter less than or equal to 2.5 μm in diameter; r-MSSD, square root of the mean squared differences of successive intervals; SDNN, standard deviation of normal-to-normal intervals.

**Table 2:** Percentage changes^a^ in heart rate variability indices for an interquartile range change of 5-minute mean PM_2.5_ in mixed-effects models.

	All workers N = 11520	With respirator N = 6528	Without respirator N = 4992
Log_10_ SDNN	−8.9	−2.2	−12.4
	(−14, 5, −3.3)	(−6.3, 1.9)	(−18.8, −6.0)
Log_10_ r-MSSD	−10.3	−4.0	−14.7
	(−15.8, −4.8)	(−6.4, −1.6)	(−20.8, −8.6)

PM_2.5_, particulate matter less than or equal to 2.5 μm in diameter; r-MSSD, square root of the mean squared differences of successive intervals; SDNN, standard deviation of normal-to-normal intervals.

^a^The values are presented as percentage changes and 95% confidence interval for interquartile range changes after adjusting for age, body mass index, smoking, temperature and relative humidity in all models.

**Table 3:** Percentage changes^a^ in heart rate variability indices for an interquartile range change of 5-minute mean PM_2.5_ in mixed-effects models stratified by smoking.

	With respirator		Without respirator	
	Smokers N = 2400	Non-smokers N = 4128	Smoker N = 1920	Non-smoker N = 3072
Log_10_ SDNN	−3.6	−1.8	−14.8	−11.6
	(−7.4, 0.2)	(−4.5, 0.9)	(−19.2, −10.4)	(−13.8, −9.4)
Log_10_ r-MSSD	−5.8	−3.1	−17.2	−12.1
	(−8.1, −3.5)	(−6.5, 0.3)	(−22.1, −12.3)	(−15.7, −8.5)

PM_2.5_, particulate matter less than or equal to 2.5 μm in diameter; r-MSSD, square root of the mean squared differences of successive intervals; SDNN, standard deviation of normal-to-normal intervals.

^a^The values are presented as percentage changes and 95% confidence interval for interquartile range changes after adjusting for age, body mass index, temperature and relative humidity in all models.

**Table 4:** Effect modification of association of heart rate variability with 5-minute mean PM_2.5_ among 120 study participants.

	Log_10_ SDNN N = 11520	Log_10_ r-MSSD N = 11520
Respirator usage		
Yes	−1.8 (−2.2, 0.4)	−3.5 (−6.8, −0.2)
No	−7.4 (−9.8, −5.0)	−10.2 (−13.7, −6.7)
P-value for interaction	0.03	0.04

BMI, body mass index; PM_2.5_, particulate matter less than or equal to 2.5 μm in diameter; r-MSSD, square root of the mean squared differences of successive intervals; SDNN, standard deviation of normal-to-normal intervals.

^a^The values are presented as percentage changes and 95% confidence interval for interquartile range changes after adjusting for age, body mass index, smoking, temperature and relative humidity in all models.
